# Differentiation between bipolar versus unipolar depression by AI‐based multimodal behavioral analysis

**DOI:** 10.1111/pcn.70124

**Published:** 2026-09-11

**Authors:** Hidenori Yamasue, Sanskar Rahul Nanegaonkar, Yumi Kiyama, Mamindla Vishwa Raghava Reddy, Kotaro Ando, Shozo Kamiya, Jun Inoue, Asuka Sakurai, Tahichi Nakano, Amane Sato, Yosuke Kameno, Tomoyasu Wakuda, Keiichi Odagiri, Tadafumi Kato

**Affiliations:** ^1^ Department of Psychiatry Hamamatsu University School of Medicine Hamamatsu Japan; ^2^ United Graduate School of Child Development Osaka University, Kanazawa University, Hamamatsu University School of Medicine, Chiba University and University of Fukui Suita Japan; ^3^ I'mbesideyou Tokyo Japan; ^4^ Center for Clinical Research Hamamatsu University Hospital Hamamatsu Japan; ^5^ Department of Child and Adolescent Psychiatry Hamamatsu University School of Medicine Hamamatsu Japan; ^6^ Hamamatsu University School of Medicine Hamamatsu Japan; ^7^ Department of Psychiatry and Behavioral Science Juntendo University Graduate School of Medicine Tokyo Japan

**Keywords:** bipolar disorder, depression, digital phenotyping, facial expressions, voice prosody

## Abstract

**Background:**

Given their differing pathophysiology and treatment approaches, the primary aim of the current study was to develop a methodology accurately distinguishing bipolar from unipolar depression.

**Methods:**

Adults with bipolar disorder or major depressive disorder were recruited nationwide in Japan and assessed *via* recorded interviews. During the interview, diagnoses were established using the Structured Clinical Interview for DSM‐5‐Research Version. Depressive and manic symptom severity was assessed using the Hamilton Depression Rating Scale (HDRS) and Young Mania Rating Scale (YMRS), respectively. Artificial intelligence (AI)‐based integrative, quantitative analyses of behavioral characteristics, including facial expressions, eye gaze, and voice prosody, were conducted on the recorded video.

**Results:**

Between August 2022 and March 2024, 379 participants completed interviews, of whom 366 were analyzed (120 bipolar I, 93 bipolar II, 122 major depressive, and 74 persistent depressive disorder). AI‐based diagnostic differentiation between bipolar and unipolar depression using four‐fold, three‐times repeated cross‐validation achieved an overall accuracy of 78%, with an area under the curve of 0.76. Key features contributing to bipolar diagnosis included greater temporal instabilities in neutral facial expression, eye gaze, sad facial expression, and root mean square amplitude of speech. HDRS could be predicted in unipolar and bipolar depression, with influential predictors of depression including sleep inefficiency, autistic traits, variability in sad facial expression, and comorbid anxiety disorder. YMRS could be predicted with major predictors including comorbid anxiety disorder, sleep inefficiency, and variabilities in sad facial expression and eye movement.

**Conclusions:**

Integrating multimodal behavioral indicators with AI‐based modeling enables clinically meaningful differentiation between bipolar and unipolar depression.

Bipolar disorder is characterized by recurring depressive and manic episodes and affects approximately 1% of the global population.^1^ However, distinguishing it from unipolar depression remains difficult, with around 40% of individuals with bipolar disorder initially misdiagnosed as having unipolar depression. Diagnostic conversion is not uncommon; 12.9% of patients first diagnosed with unipolar depression are later reclassified as bipolar disorder,^2^ with an average delay of 10.8 years.^3^ Such delays are problematic because treatment strategies differ markedly between the two conditions, and prescribing antidepressants intended for unipolar depression can worsen symptoms in bipolar disorder, increasing manic risk^4^ and contributing to greater social impairment and suicidality.^5^ Early and accurate differentiation is therefore essential.

Misdiagnosis often arises from overlapping depressive symptomatology. Because patients usually seek help during depressive episodes^6^ and may underreport or fail to recognize hypomanic symptoms,^7^ clinicians face difficulty distinguishing the two disorders based solely on depressive presentation.^8^ Although certain clinical features—such as hypersomnia, mood instability, impulsivity, irritability, emotional reactivity, psychomotor activation, increased appetite, early onset, antidepressant resistance, and family history—have been proposed to differentiate bipolar from unipolar depression,^9^, ^10^, ^11^, ^12^ these indicators rely heavily on subjective reporting and variable clinical judgment.

Although verbally reported experiences, such as depressed mood and diminished interest or pleasure, are central to the description of diagnostic criteria, nonverbal behaviors—including facial expressions, speech characteristics, gaze patterns, and gestures—are clinically important indicators of an individual's mental state and diagnosis. Recent studies incorporating AI‐based objective assessments have further supported the importance of such objective behavioral measures, beyond verbal descriptions alone, in characterizing psychiatric conditions.^13^ Recent research has explored digital phenotyping to improve diagnostic accuracy. A recent systematic review^14^ summarized 21 studies, though many involved remitted patients rather than direct comparisons between active unipolar and bipolar depression. Studies focusing on this distinction^15^, ^16^, ^17^ typically analyzed only one behavioral modality—such as facial expressions or speech—and were limited by small sample sizes (23‐169 participants). Many lacked independent validation samples and used LOOCV, resulting in limited discriminative power.^16^ Although these studies highlight the promise of digital phenotyping, reproducibility and AUC values remain modest.

To address these limitations, the present study applied artificial intelligence (AI) to quantify multiple behavioral and physiological parameters—facial expressions, speech prosody, eye movements, and sleep patterns—in a large sample of individuals with bipolar disorder and unipolar depression. Symptoms characteristic of manic states, such as euphoria, psychomotor agitation, irritability, heightened emotional reactivity, increased talkativeness, pressured speech, and decreased need for sleep, as well as symptoms characteristic of depressive states, including psychomotor retardation, depressed mood, loss of interest or pleasure, feelings of sadness, and insomnia or hypersomnia,^13^, ^18^, ^19^, ^20^ are clinically reflected in changes in facial expressions of emotion, speech prosody, speech quantity and rate, eye‐gaze behavior, and sleep‐related variables. By integrating these multimodal data with clinical information through machine learning, the primary aim of the current study was to develop an objective diagnostic support system distinguishing bipolar from unipolar depression, and the secondary aim was to predict depressive and manic symptom severities with such a system.

## Methods

### Study design and participants

This cross‐sectional observational study included participants with bipolar disorder or unipolar depression, who were assessed through video interviews (Pre‐registered: UMIN000048854). Participants were recruited through advertisements posted on websites and posters displayed in outpatient clinics across 170 hospitals in Japan. All participants received a full explanation of the study procedures and provided written informed consent. The authors assert that all procedures contributing to this work comply with the ethical standards of the relevant national and institutional committees on human experimentation and with the Helsinki Declaration of 1975, as revised in 2013. All procedures involving human subjects/patients were approved by the Ethics Committee of Hamamatsu University School of Medicine (21‐335). Inclusion criteria were as follows: (i) age ≥18 years, and (ii) a clinical diagnosis of either bipolar disorder or major depressive disorder.

### Diagnosis and clinical assessments

After providing informed consent, participants completed a questionnaire to collect background information including age, sex, clinical diagnosis, treatment history, and medication history. Eligible participants were then interviewed *via* Zoom (Zoom Communications, Inc.).

During the video interview, a trained psychiatrist or psychologist determined each participant's diagnosis using the Structured Clinical Interview for DSM‐5 Research Version (SCID‐5‐RV).^21^ The severity of depressive and manic symptoms was assessed using the Hamilton Depression Rating Scale (HDRS)^22^ and the Young Mania Rating Scale (YMRS),^23^ respectively. The prevalence of both comorbid bipolar disorder and unipolar depression is substantially higher in autism spectrum disorder (ASD) than in the general population.^24^, ^25^ Furthermore, because previous studies have demonstrated significant associations between autistic traits and facial expressions,^26^, ^27^, ^28^ voice prosody,^29^, ^30^, ^31^ and eye gaze,^32^ autistic traits were also evaluated using the Autism‐Spectrum Quotient (AQ).^33^ To facilitate emotional expression during the conversation, the Autism Diagnostic Observation Schedule (ADOS)^34^ emotion activity was also administered during the same Zoom interview based on the justification in our previous study.^26^

### Feature extraction

The entire Zoom session recording was used as input. Facial and vocal features were extracted on a frame‐wise basis from the full session, and these frame‐level features were subsequently aggregated to generate the final statistical features (e.g., mean across all frames, standard deviation (SD), and SD of SD). No specific interview sub‐segments were selected; rather, the analysis encompassed the entire duration of each session (Fig. 1). Recorded interview videos were processed using a custom computational pipeline to extract several low‐level behavioral and physiological attributes, including: Eye gaze–related expressions including blink rate, eye offset, and gaze^35^, ^36^; Facial expressions including anger, disgust, fear, happiness, sadness, surprise, and neutral^37^, ^38^; Voice features^39^, ^40^ (Fig. 2) (see details in Data S1).

**Fig. 1:**
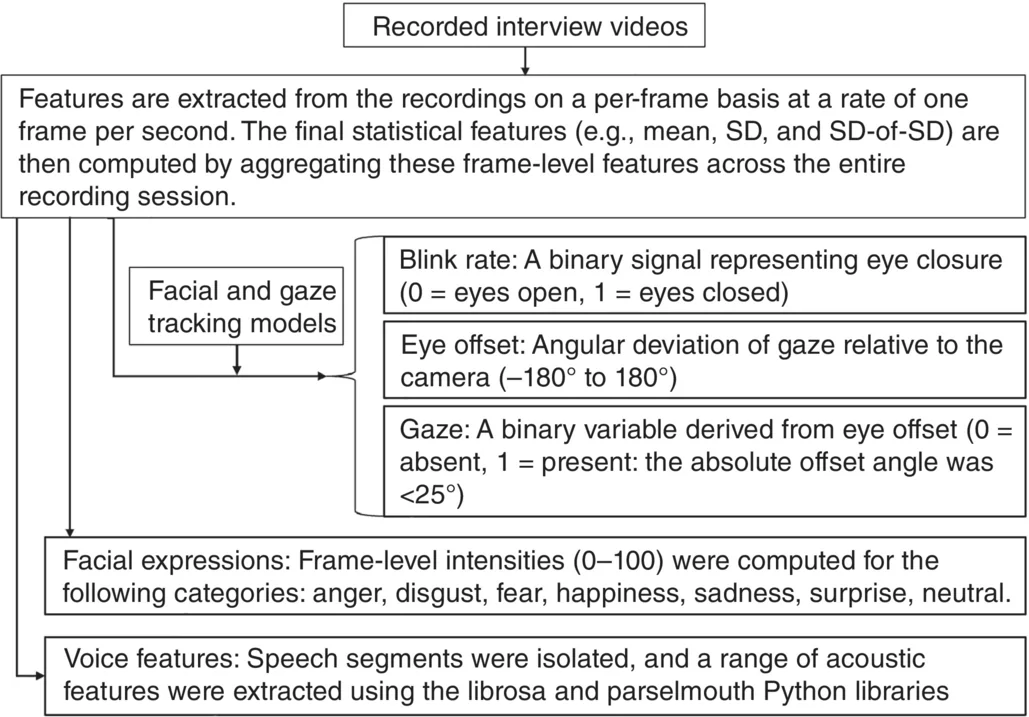
AI feature‐extraction workflow.

**Fig. 2:**
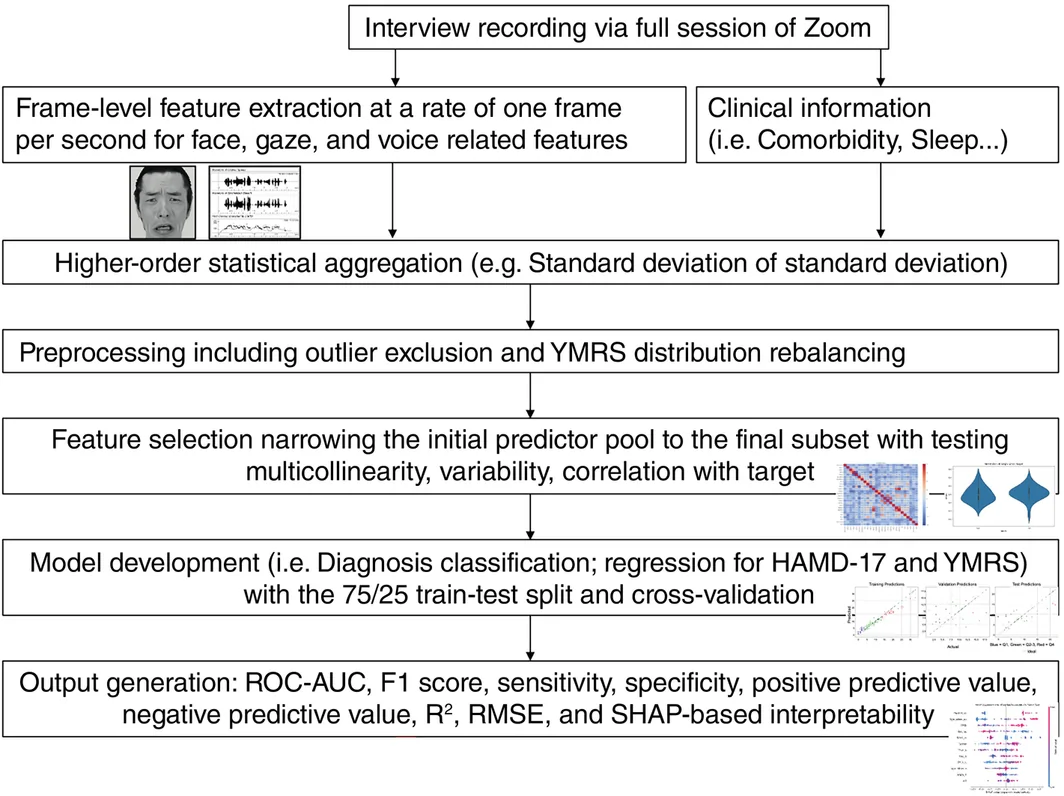
Data‐processing architecture flow.

### Statistical feature derivation

To summarize temporal dynamics of visual and acoustic signals, a set of higher‐order statistical descriptors was calculated: Standard Deviation (s): Quantifies variability in feature values; Standard Deviation of Standard Deviation (ss): Represents temporal instability, calculated as follows: (i) divide each feature's time series into non‐overlapping 30‐s intervals; (ii) compute the standard deviation within each interval; (iii) calculate the standard deviation across all interval‐level standard deviations. A higher value indicates greater temporal variability within the session.

Facial and vocal features were treated similarly to previous approaches, alongside additional predictors that integrated diagnostic information from SCID‐5 and sleep‐related features extracted from HDRS and YMRS, such as recent and habitual records of bedtime, sleep onset time, awakening time, and get‐up time. Using clinician‐rated variables derived from the interview to predict clinician‐assigned diagnoses could artificially inflate model performance. Therefore, clinician‐rated variables directly related to mood disorders were not included as predictive features; only the presence or absence of comorbid diagnoses established using SCID was included among the predictors.

### Sample size calculation

The target sample size was estimated using power analysis, resulting in a minimum total sample size of 220. However, since the analyses in this study were exploratory, and given that the number of predictors and analytical design could change, data collection continued beyond this minimum target to obtain as many cases as possible within the data collection period (until March 2024) (See details in Data S1).

### Data preprocessing

All recorded data were pre‐processed prior to analysis. The dataset was randomly split into training, validation, and test subsets using ratios of 70%–15%–15%, or training and test sets of 75%–25% for smaller samples, while maintaining approximately equal distributions of target variables across splits. Potential outliers were also excluded to stabilize the model training. (See details including the definition of ground truth labels, the justification for preprocessing steps such as outlier exclusion and YMRS subsampling in Data S1).

### Feature preparation

A total of 42 behavioral and clinical features were used, including facial, vocal, and gaze‐related indices, as well as sleep‐related and clinical questionnaire measures. Multicollinearity among features was examined, and redundant variables were removed (|*r*| > 0.8). Features with minimal variability (variance <0.01) or very low correlation with target variables (*r* < 0.01) were excluded. The distributions and relationships between individual features and outcome variables were visually inspected using scatter and violin plots to guide feature retention decisions. Feature selection was conducted *via* Recursive Feature Elimination with Cross‐Validation using the Extreme Gradient Boosting (XGBoost) classifier, optimized for the mean F1 score across folds. (See details in Data S1).

### Model training and validation

For the classification task differentiating unipolar and bipolar depression, bagging classifiers wrapped in a calibrated classifier (isotonic calibration) were trained using four‐fold, three‐times repeated cross‐validation. The final prediction was made using a weighted ensemble model combining 12 bagging classifiers derived from cross‐validation folds.

For the regression tasks predicting depressive or manic symptom severity, several machine learning models were trained, including CatBoost, ExtraTrees, RandomForest, and Ridge Regression. Hyperparameter tuning was performed using a composite metric defined as RMSE/[1 + *R* ^2^] to balance bias and variance. A VotingRegressor ensemble was constructed by integrating the three best‐performing models (and, in some cases, an additional Ridge model as a linear component). Model performance was evaluated on training, validation, and unseen test datasets.

Subgroup analyses were conducted stratified by sex and, for bipolar participants, by bipolar I *vs*. II subtype. (See details in Data S1).

### Model evaluation and interpretability

Model performance metrics included accuracy, receiver operating characteristic curve (ROC‐AUC), F1 score, sensitivity, specificity, positive predictive value (PPV), negative predictive value (NPV), *R* ^2^, and root‐mean‐squared error (RMSE), representing the average magnitude of the difference between the predicted and the actual values, depending on the analytic task. Calibration of probabilistic outputs was evaluated using calibration plots, with model reliability assessed across probability bins. Model interpretability was assessed using SHapley Additive exPlanations (SHAP) analysis (See details in Data S1).

## Results

### Participants

The data collection was conducted from August 2022 to March 2024. Among the 505 subjects who provided written consent and participated in the study, 459 subjects were confirmed as eligible, and 379 participants completed the Zoom interview. Based on the lifetime diagnosis of SCID‐5, 120 was diagnosed as bipolar disorder type 1, 93 as bipolar disorder type 2, 122 as major depressive disorder, and 74 has diagnosis of continuous depressive disorder, while 25 participants did not meet criteria for any of these diagnoses. The overlap with major depressive disorder and continuous depressive disorder was recognized in 55 participants. 366 of the 379 participants were analyzed, since the 13 participants were excluded because of the incomplete recordings. No significant diagnostic‐group differences were observed in age, sex distribution, educational level, marital status, or employment status (all *P* > 0.10). Significant diagnostic‐group differences were observed for current mood episode status, HAMD‐17 scores, and YMRS scores (all *P* < 0.05). These differences were primarily attributable to the inclusion of participants experiencing current manic or hypomanic episodes exclusively within the bipolar disorder groups, higher HAMD‐17 scores in participants with major depressive disorder and persistent depressive disorder, and higher YMRS scores in participants with bipolar disorder type I and lower YMRS scores in those with major depressive disorder (Table 1, Fig. S1).

**Table 1:** Demographic and clinical characteristics of participants

Lifetime diagnosis	Bipolar disorder		Major depressive disorder	Continuous depressive disorder	Differences by diagnoses	
	Type 1: *N* = 120	Type 2: *N* = 93	*N* = 122	*N* = 74	*F* or χ^2^	*P*‐value
Age (y.o.)	40.1 (12.9)	36.8 (12.4)	40.2 (12.9)	41.1 (13.5)	*F* = 1.42	0.24
Sex: Male/Female/Others	46/74/0	24/68/1	40/80/2	27/47/0	χ^2^ = 6.13	0.41
Highest level of education (1–8^†^, %)	0/1.7/21.7/9.2/15.0/38.3/3.3/10.8	0/5.4/22.6/8.6/21.5/31.2/1.1/9.7	0/0.8/19.7/4.1/19.7/45.9/0/9.8	0/1.4/21.6/4.1/24.3/37.8/0/10.8	χ^2^ = 19.25	0.38
Marital status (1–5^‡^, %)	35.0/0/14.2/0.8/50.0	38.7/0/8.6/0/52.7	41.0/0.8/13.9/0.8/43.4	29.7/0/14.9/1.4/54.1	χ^2^ = 10.59	0.56
Employment status (1–8^§^, %)	32.5/28.3/3.3/5.8/5.0/11.7/3.3/6.7	33.3/34.4/1.1/10.8/2.2/8.6/4.3/4.3	43.4/33.6/3.3/4.1/3.3/4.1/4.9/3.3	29.7/36.5/4.1/5.4/4.1/4.1/6.8/9.5	χ^2^ = 29.49	0.1
The current episode: Manic/Hypomanic/Depression/Remission	14/4/19/83	0/15/24/54	0/0/52/70	0/0/46/28	χ^2^ = 252.27	<0.001
HAMD17 score	7.92 (6.51)	7.92 (6.15)	10.5 (7.40)	11.1 (7.59)	*F* = 3.67	0.013
YMRS score	4.49 (5.48)	3.04 (4.31)	2.47 (3.80)	3.12 (4.58)	*F* = 4.44	0.004

^†^ Highest level of education: (i) Elementary school; (ii) Junior high school; (iii) High school; (iv) Dropout from University; (v) College or vocational school; (vi) University; (vii) Dropout from graduate school; (viii) Garuate school.

^‡^ Marital status: (i) Married or common‐law marriage; (ii) Bereavement; (iii) Divorce; (iv) Separation; (v) Unmarried.

^§^ Employment status: (i) Full‐time employment; (ii) Part‐time employment; (iii) Full‐time housework or caregiving; (iv) Currently studying or in training; (v) Retired; (vi) Unemployed and seeking work; (vii) Unemployed and not seeking work; (viii) Disabled.

### Differentiation between unipolar and bipolar depression

Patients were first filtered based on whether they were experiencing a current or most recent depressive episode. A total of 91 participants with a current depressive episode were analyzed, of whom 42 had a lifetime diagnosis of bipolar disorder and 49 had a lifetime diagnosis of unipolar depression. Because of the limited sample size, the dataset was divided into training and test sets in a 75–25% ratio.

After feature selection among the 42 candidate features, the optimal features identified for each fold are presented in Fig. S2. The analyses revealed that the differences between the two types of depression were best explained by a specific set of 12 features: ss of the prevalence of neutral facial expression (Neutral_ss), ss of eye offset (Eye_offset_ss), s of eye offset (Eye_offset_s), root mean square of speech (RMS), tempo of speech (Tempo), s of pitch of speech (Pitch_s), ss of sad facial expression (Sad_ss), s of sad facial expression (Sad_s), mean of eye blink (Blink_m), s of fearful facial expression (Fear_s), s of angry facial expression (Angry_s), and autistic tendency (AQ). The overall accuracy of the diagnostic model was approximately 78%, with accuracies of 67% for unipolar and 91% for bipolar depression. The ROC‐AUC was 76%. The F1 score was 78%. The sensitivity was 91%, specificity was 67%, PPV was 71%, and NPV was 89%. Subgroup analyses stratified by sex and bipolar subtype yielded comparable results as follows: In the test data: male (*n* = 8), accuracy = 75%, ROC‐AUC = 75%, F1 = 77%; female (*n* = 15), accuracy = 80%, ROC‐AUC = 76%, F1 = 80%; bipolar I (*n* = 6), accuracy = 83%, F1 = 91%; bipolar II (*n* = 5), accuracy = 100%, F1 = 100%.

### Model interpretability

Notably, the Neutral_ss (mean |SHAP| = 15.1%, SD = 3.0%), Eye_offset_ss (mean |SHAP| = 14.2%, SD = 7.2%), RMS (mean |SHAP| = 11.0%, SD = 4.8%), and Sad_ss (mean |SHAP| = 10.4%, SD = 5.2%) emerged as the most influential contributors, showing higher values in bipolar depression compared with unipolar depression (Fig. 3).

**Fig. 3:**
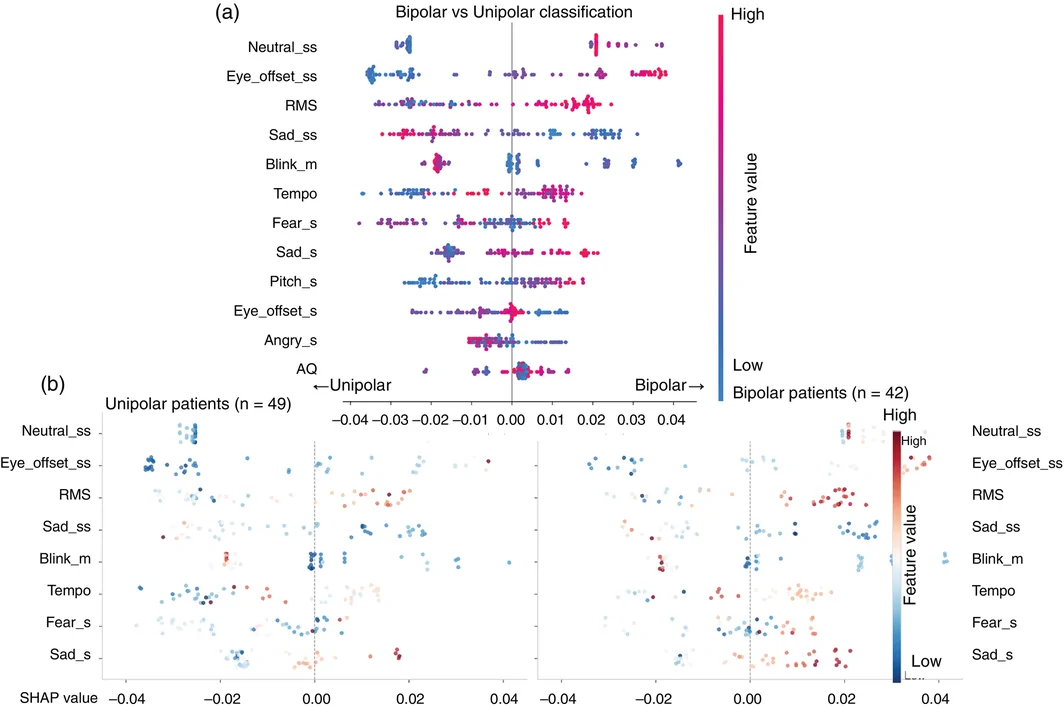
SHAP plots for the differentiation between unipolar and bipolar depression. (a) SHAP beeswarm plot on train and test differentiation between unipolar and bipolar depression showing the features importance for the differentiation between unipolar and bipolar depression. The *x* axis represents the features' contribution to the final prediction, with positive or negative values associated with an increase in the likelihood of a bipolar or unipolar diagnosis, respectively. The *y* axis lists the features in descending order of importance. The blue–red color gradient indicates the relevance of each of the features to the score, from low to high values. For each feature, a point represents the normalized SHAP value of an individual participant. (b) The diagnosis‐specific beeswarm plots for unipolar patients (left) and bipolar patients (right), respectively.

### Calibration

The curve generally aligns with the perfect calibration line, particularly in the mid‐ to high‐probability ranges (Fig. S3). However, some deviation is observed in the fifth bin (0.80–1.00). This bin contains only two data points, making it less reliable for evaluating perfect calibration. Despite the small sample size, the fraction of positives is slightly underpredicted. Overall, the model demonstrates improved calibration as the predicted probabilities increase, particularly from the mid‐ to high‐confidence bins.

### Prediction of depressive/manic symptoms

The analysis for predicting depressive symptom severity was conducted separately in patients with unipolar depression and those with bipolar disorder based on the current diagnosis and the classifying model, given the differing manifestations of depressive symptoms between these diagnostic groups. The unipolar dataset included all available participants classified as having unipolar depression (*n* = 156), whereas the bipolar dataset included all available participants classified as having bipolar disorder (*n* = 200). Each dataset was split into training, validation, and test sets in a 70%–15%–15% ratio, ensuring an equal distribution of the target variable across splits.

### Prediction of depressive symptoms in unipolar depression

Among the 42 candidate features, for the unipolar group, the final selected features, based on the above analyses, included: AQ, Sad_s, local jitter of voice (Local_Jitter), Happy_s, Sad_ss, Eye_offset_ss, sleep inefficiency during the last 1 week (Sleep_inefficiency_recent), difference in recent sleep efficiency relative to the usual pattern (Change_in_sleep_efficiency), and the current diagnosis of anxiety disorders (Anxiety_Diagnosis). The model performance was as follows: Training set: *R* ^2^ = 0.92; RMSE = 1.98; Validation set: *R* ^2^ = −0.35; RMSE = 5.39; Test (unseen) data: *R* ^2^ = 0.36; RMSE = 4.97 (Fig. 4b). Subgroup analyses stratified by sex showed comparable results as follows: In the test data: Male (*n* = 7): *R* ^2^ = 0.2448; RMSE = 6.1272; Female (*n* = 15): *R* ^2^ = 0.4277; RMSE = 4.6732.

**Fig. 4:**
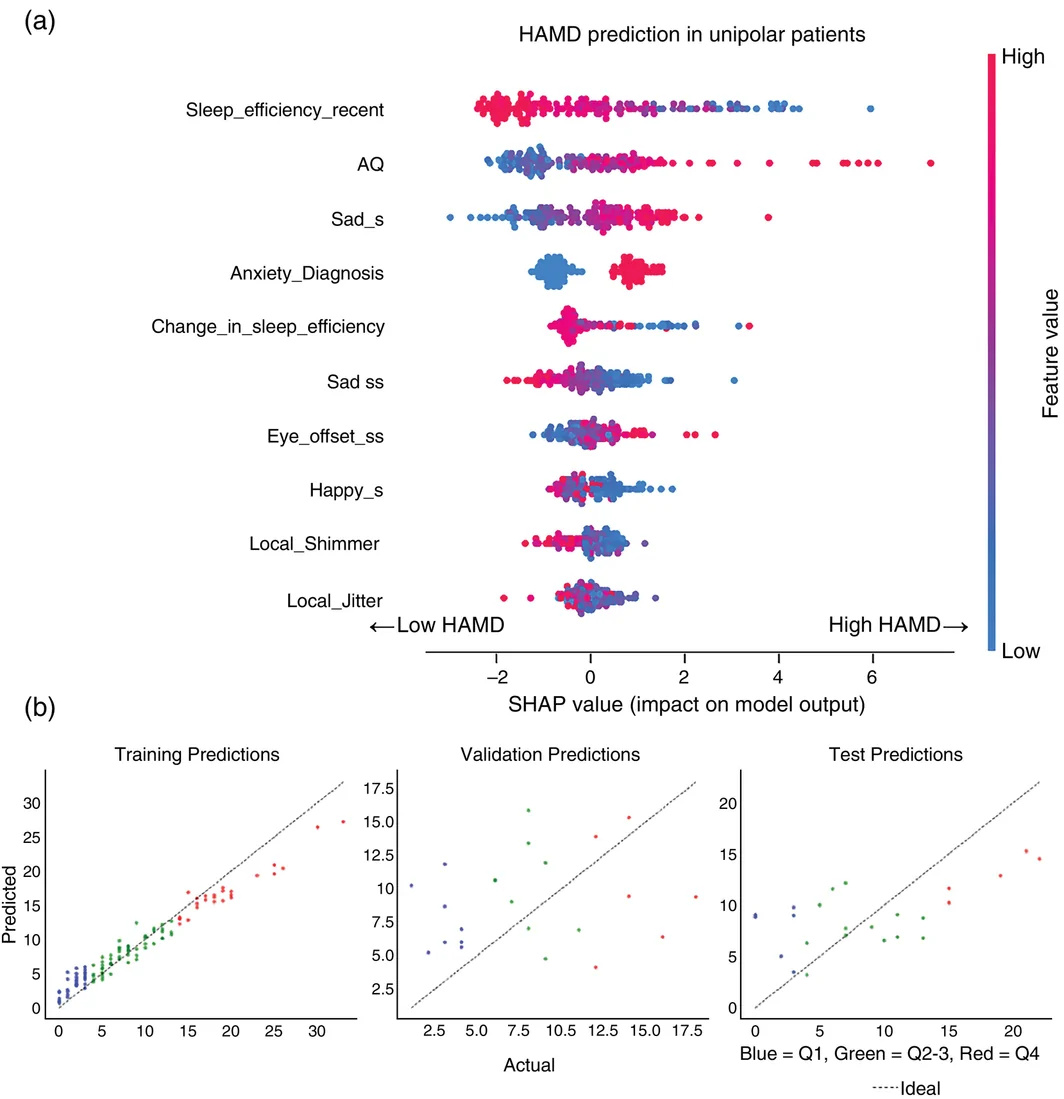
Prediction of depressive symptom severity in unipolar depression: (a) SHAP beeswarm plot showing the features importance for the prediction of HDRS score. The *x* axis represents the features' contribution to the final prediction, with positive or negative values associated with an increase in the HDRS score. The *y* axis lists the features in descending order of importance. The blue–red color gradient indicates the relevance of each of the features to the score, from low to high values. For each feature, a point represents the normalized SHAP value of an individual participant. (b) Scatter plots for predictions of HDRS scores: Training set (Left): *R* ^2^ = 0.92; RMSE = 1.98; Validation set (Center): *R* ^2^ = −0.35; RMSE = 5.39; Test (unseen) data (Right): *R* ^2^ = 0.36; RMSE = 4.97.

### Model interpretability

The most influential predictors included Sleep_inefficiency_recent (mean|SHAP| = 21.2%, SD = 15.0%), AQ (mean|SHAP| = 17.1%, SD = 16.7%), Sad_s (mean|SHAP| = 17.9%, SD = 8.7%), and Anxiety_Diagnosis (mean|SHAP| = 11.7%, SD = 3.3%). All these features were positively associated with higher HDRS scores, indicating that greater impairment in these domains predicted greater depressive symptom severity (Fig. 4a).

### Prediction of depressive symptoms in bipolar depression

The bipolar dataset consisted of 200 participants. The data were split into training, validation, and test sets in a 70%–15%–15% ratio, ensuring an equal distribution of the target variable across all splits.

The following features were selected for the bipolar dataset: AQ, Sad_s, harmonic to noise ratio of speech voice (HNR), Angry_ss, Blink_s, Standard Deviation of the negative (Angry + Disgust + Fear + Sad) facial expression (Negative_s), Local_Jitter, RMS, Gaze_ss, Negative_ss, Sleep_efficiency_recent, Sleep_efficiency_usual, Change_in_sleep_efficiency, Anxiety_Diagnosis, and Angry_s.

A VotingRegressor ensemble was then constructed using the three best‐performing models overall, as well as the best‐performing models within the higher and lower HDRS score ranges. The model performance was as follows: Training set: *R* ^2^ = 0.91; RMSE = 1.87; Validation set: *R* ^2^ = 0.22; RMSE = 5.45; Test (unseen) data: *R* ^2^ = 0.11; RMSE = 5.44 (Fig. 5b). Subgroup analyses stratified by sex showed broadly comparable results as follows: In the test data: Male (*n* = 11): *R* ^2^ = −0.1428; RMSE = 5.2762; Female (*n* = 19): *R* ^2^ = 0.2038; RMSE = 5.5528.

**Fig. 5:**
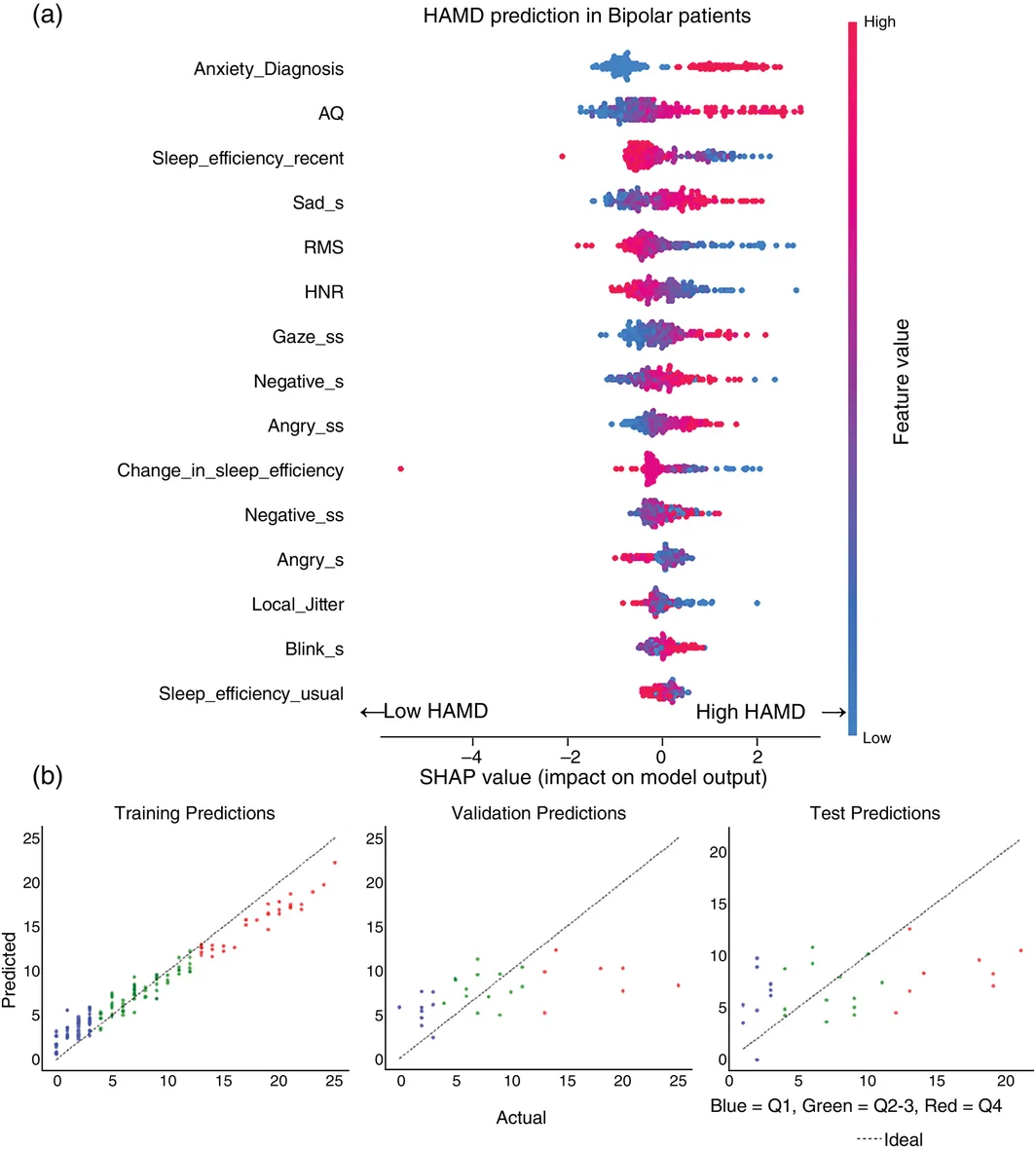
Prediction of depressive symptom severity in bipolar dataset. (a) SHAP beeswarm plot showing the features importance for the prediction of HDRS score. The *x* axis represents the features' contribution to the final prediction, with positive or negative values associated with an increase in the HDRS score. The *y* axis lists the features in descending order of importance. The blue–red color gradient indicates the relevance of each of the features to the score, from low to high values. For each feature, a point represents the normalized SHAP value of an individual participant. (b) Scatter plots for predictions of HDRS scores: Training set (Left): *R* ^2^ = 0.91; RMSE = 1.87; Validation set (Center): *R* ^2^ = 0.22; RMSE = 5.45; Test (unseen) data (Right): *R* ^2^ = 0.11; RMSE = 5.44.

### Model interpretability

The most influential predictors included Anxiety_Diagnosis (mean|SHAP| = 15.2%, SD = 6.6%), AQ (mean|SHAP| = 12.8%, SD = 9.8%), Sleep_inefficiency_recent (mean|SHAP| = 8.8%, SD = 6.3%), and Sad_s (mean|SHAP| = 8.5%, SD = 6.1%). Higher values of these features were associated with higher HDRS scores, indicating their significant contribution to predicting depressive symptom severity in bipolar depression (Fig. 5a).

### Prediction of manic symptoms

All patients were included regardless of their current diagnosis, as no *a priori* hypothesis was established concerning manic characteristics in patients with unipolar depression. Due to an excessive number of data points in the lower quartiles of the target variable, several preprocessing adjustments were made: (1) 60% of zero values were randomly removed from the training set and evaluated separately; (2) 20% of data points within the range of 1–6 were randomly removed and tested separately; and (3) potential outliers were excluded. The full dataset consisted of 349 samples and was split into training, validation, and test sets in a 70%–15%–15% ratio. After handling the excessive low‐end data points, the final sample sizes were: training = 166, validation = 53, test = 52, and additional test (from the excluded training points) = 78. Each split maintained an approximately equal distribution of the target variable.

The selected features were: Anxiety_Diagnosis, s of pitch of speech (Pitch_s), Eye_offset_s, Sad_s, Sleep_efficiency_recent, average sleep time over the past week minus the usual average sleep time (Change_in_sleep_time), Happy_ss, and Local_Jitter. Based on the validation results, we could observe that the CatBoost and ExtraTrees Regressor are performing the best and observing the upper quartile scores, ExtraTreesRegressor stands out as the best tree model. YMRS Analysis after Handling Lower‐Range Data showed the model performance as follows: Training set: *R* ^2^ = 0.73; RMSE = 2.60; Validation set: *R* ^2^ = −0.12; RMSE = 4.88; Test (unseen) data: *R* ^2^ = 0.05; RMSE = 3.99 (Fig. 6b). Subgroup analyses stratified by sex yielded comparable results as follows: In the test data: Male (*n* = 16): *R* ^2^ = −1.2421; RMSE = 2.5664; Female (*n* = 36): *R* ^2^ = 0.0888; RMSE = 4.4844.

**Fig. 6:**
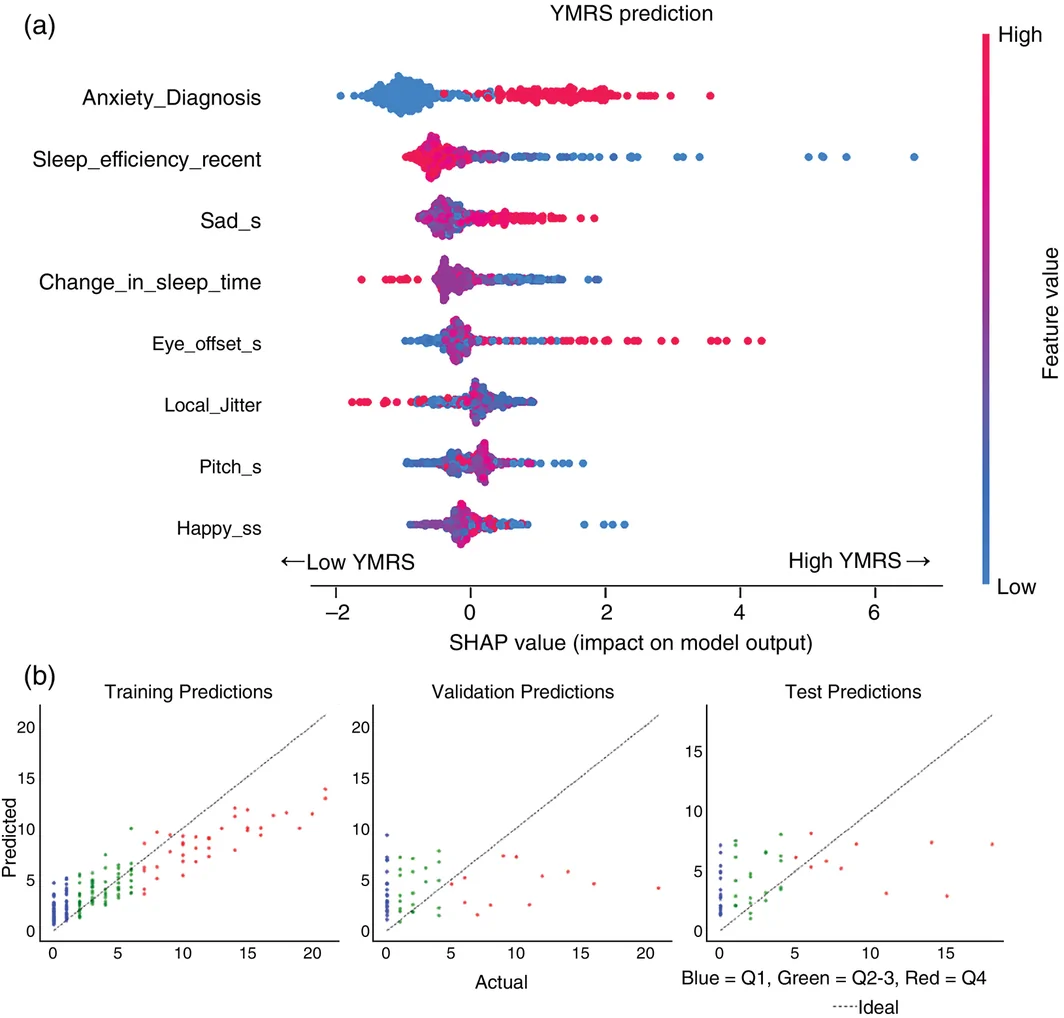
Prediction of manic symptom severity. (a) SHAP beeswarm plot showing the features importance for the prediction of YMRS score. The *x* axis represents the features' contribution to the final prediction, with positive or negative values associated with an increase in the YMRS score. The *y* axis lists the features in descending order of importance. The blue–red color gradient indicates the relevance of each of the features to the score, from low to high values. For each feature, a point represents the normalized SHAP value of an individual participant. (b) Scatter plots for predictions of YMRS scores: Training set (Left): *R* ^2^ = 0.73; RMSE = 2.60; Validation set (Center): *R* ^2^ = −0.12; RMSE = 4.88; Test (unseen) data (Right): *R* ^2^ = 0.05; RMSE = 3.99.

### Model interpretability

The most influential predictors included Anxiety_Diagnosis (mean|SHAP| = 30.6%, SD = 12.1%), Sleep_inefficiency_recent (mean|SHAP| = 16.0%, SD = 11.8%), Sad_s (mean|SHAP| = 11.2%, SD = 6.5%), and Eye_offset_s (mean|SHAP| = 10.2%, SD = 10.9%). Higher values of these features were associated with higher YMRS scores, suggesting their strong contribution to predicting manic symptom severity (Fig. 6a).

## Discussion

The present study used integrative AI analyses of digital phenotyping—including facial expressions, voice prosody, eye‐gaze, sleep‐related data, and clinical information—to objectively differentiate bipolar from unipolar depression and to predict depressive and manic symptom severity. Using a world dataset, we achieved reliable diagnostic classification and symptom prediction, with performance largely unaffected by sex or diagnostic subtype. The analyses also identified key behavioral and clinical features contributing to these outcomes.

Our integrative approach demonstrated high discriminative performance (AUC: 78%; ACC: 76%), exceeding that of many previous studies, which reported more modest results (AUC: 43–79%; ACC: 50–76%).^15^, ^16^, ^41^, ^42^, ^43^ Earlier work often analyzed each modality in isolation, whereas combining behavioral expression data with clinical indices and sleep characteristics likely improved performance. This multimodal integration may mimic clinicians' natural information processing, contributing to higher accuracy.

Compared with the diagnosis discrimination, the performance for prediction of manic (RMSE: 3.99 for the test set) and depressive severity (RMSE: 5.44 for the test set) was limited. Based on the hypothesis that bipolar and unipolar depression exhibit different behavioral signatures, we first classified the diagnostic groups and then predicted symptom severity within each group based on the clinical understandings of differing pathophysiology.^44^ However, given the limited performance especially for the prediction of manic symptoms, the most likely reason is the limited number of high‐YMRS samples in the dataset, which restricts how well the model can learn behavioral patterns associated with elevated manic symptomatology. Future studies should test datasets including a sufficient number of participants with high YMRS scores.

SHAP analyses revealed several clinically meaningful features. Greater temporal variabilities in neutral facial expression, eye movements, and sad facial expression, along with higher vocal loudness and acoustic energy, were associated with bipolar depression, consistent with prior descriptions of emotional reactivity, agitation, and psychomotor activation.^10^, ^12^ In both diagnostic groups, poor sleep efficiency, sad facial expressions, and anxiety contributed to depressive severity, consistent with established clinical observations.^44^ In bipolar depression, autistic traits were more strongly linked to both manic and depressive symptoms, and eye‐movement features were particularly relevant for predicting manic symptoms—findings in line with reports of ASD comorbidity^45^ and characteristic ocular patterns in bipolar disorder.^43^ Importantly, these results were obtained from data collected in diverse real‐world environments (indoors and inside a parked vehicle, with varied lighting and camera types). The stable performance across heterogeneous conditions suggests strong potential for clinical implementation.

Several limitations merit consideration. First, participants were native Japanese speakers, and nonverbal expressions vary across cultures^46^; further cross‐cultural validation is needed. Second, the proportion of bipolar depression cases was higher than in general clinical settings, indicating the need for studies with more representative diagnostic distributions. Third, although the present study analyzed data from a relatively large overall sample (*n* = 366), the primary diagnostic classification analysis was conducted in a smaller subgroup (*n* = 91). Especially, the subgroup analyses were particularly underpowered (e.g., bipolar II disorder, *n* = 5 in the test dataset). Therefore, the findings should be interpreted with appropriate caution and require validation in future studies with larger samples. Subtype‐specific differences (bipolar I vs. bipolar II) require larger samples for stratified analyses. Fourth, some individuals initially diagnosed with unipolar depression may later convert to bipolar disorder,^2^ highlighting the importance of longitudinal follow‐up. Given the limited sample size and the cross‐sectional nature of this study, the findings should be interpreted with caution, particularly with regard to the potential for overfitting, the influence of preprocessing decisions, and the absence of independent external validation. Fifth, the use of more objective sleep measures, such as actigraphy or mobile monitoring devices,^47^ may improve discriminative accuracy. Overall, we should frame the current findings as preliminary and hypothesis‐generating rather than implementation‐ready.

As an additional methodological consideration, the final model was not a single classifier trained on all 42 candidate predictors. Rather, the initial set of 42 predictors was first reduced through feature‐selection procedures to a smaller subset of approximately 12 features. The final model consisted of an ensemble of bagging classifiers, with each base classifier trained on a different subset of these selected features. Feature subsets and model types were optimized through Optuna‐based hyperparameter searches across multiple feature combinations. Consequently, no individual base classifier was trained on the full set of selected features. Instead, the ensemble aggregated predictions from multiple lower‐dimensional models, thereby reducing the effective model complexity of each learner and mitigating the risk of overfitting. Figure 2 illustrates the model architecture and the feature subsets selected for each fold. Nevertheless, given the relatively small sample size used for the primary classification analysis, the possibility of overfitting cannot be completely excluded and should be considered when interpreting the findings.

The present study was not designed to determine whether objective quantitative assessments of behavioral expression are superior to self‐reported measures for evaluating symptom severity. Rather, objective behavioral measures and self‐reported assessments likely capture different aspects of psychopathology and may therefore play complementary roles in clinical evaluation. With regard to the differential diagnosis of bipolar *versus* unipolar depression, objective quantitative assessments of behavioral expression may potentially provide advantages over self‐report‐based measures. However, evaluating this possibility would require prospective longitudinal studies comparing the ability of these approaches to predict subsequent conversion to bipolar disorder among individuals presenting with a depressive episode. The aim of the present study was to investigate whether behavioral expressions observed during a current depressive episode contain information indicative of a past history of hypomanic or manic episodes, thereby facilitating the differentiation of bipolar and unipolar depression. The study was not intended to assess whether AI could replace clinical judgment in diagnosing current depressive episodes.

In conclusion, this study demonstrates that integrating multimodal behavioral data with clinical information through AI enables reliable differentiation between bipolar and unipolar depression and accurate prediction of symptom severity. Using, real‐world data, these findings provide strong support for the clinical applicability of AI‐assisted diagnostic assessment.

## Author contributions

Concept and design: H.Y., S.K., T.K. Acquisition, analysis, or interpretation of data: All authors. Drafting of the manuscript: H.Y. Critical review of the manuscript for important intellectual content: S.R.N., K.A., S.K., T.K. Statistical analysis: H.Y., S.R.N., M.V.R.R., K.A., S.K. Obtained funding: H.Y. Administrative, technical, or material support: Y.K., A.S., T.N., A.S.

## Funding statement

This work was supported by the Japan Agency for Medical Research and Development (23dk0307110h0002). Neither the funder nor sponsor had any involvement in the data collection, analyses, writing, or interpretation of the study.

## Disclosure statement

Mr. Sanskar Rahul Nanegaonkar, Mamindla Vishwa Raghava Reddy, Kotaro Ando, and Shozo Kamiya are full‐time employees of *I'mbesideyou*. The other authors have no financial relationships with commercial interests. Tadafumi Kato is the Editor‐in‐Chief and Hidenori Yamasue is an Editorial Board member of Psychiatry and Clinical Neurosciences and a co‐author of this article. To minimize bias, they were excluded from all editorial decision‐making related to the acceptance of this article for publication.

## Transparency declaration

The corresponding author had full access to all the data in the study and takes responsibility for the integrity of the data and the accuracy of the data analysis.

## Supporting information


**Figure S1.** Participants flow.
**Figure S2.** Trial scores for selected features.
**Figure S3.** Calibration for differentiation of bipolar from unipolar depression.
**Table S1.** Future list and their explanations.

**Data S1.** Supporting Information.

## Data Availability

Individual‐level data or Research Material are protected and are not publicly available in a public repository. Data and the analytic code will be made available to investigators who submit a methodologically sound proposal to the corresponding author (yamasue@hama-med.ac.jp) and whose proposed use of the data has been approved by an independent review committee established for this purpose. The data are not publicly available due to them containing information that could compromise the privacy or consent of research participants. Explicit consent to deposit video data was not obtained from the participants.
