# Supplementary material for: Differentiation between bipolar versus unipolar depression by AI‐based multimodal behavioral analysis
Source: Psychiatry Clin Neurosci. 2026 Sep 11;80(11):925–34. doi: 10.1111/pcn.70124 (PMC13732806; doi:10.1111/pcn.70124)
Supplement: Supplementary file 2 [file PCN-80-925-s002.docx]

**Supplemental Information for Yamasue et al.**

**“Differentiation between Bipolar versus Unipolar Depression by AI-Based Multimodal Behavioral Analysis”**

**Supplemental Methods**

**Supplemental Figures 1-3.**

**Supplemental Table 1.**

**Supplementary methods**

**Feature Extraction**

Recorded interview videos were processed using a custom computational pipeline to extract several low-level behavioral and physiological attributes, including: Blink rate: A binary signal representing eye closure (0 = eyes open, 1 = eyes closed).; Eye offset: Angular deviation of gaze relative to the camera, estimated using facial and gaze tracking models (1, 2), with values ranging from −180° to 180°.; Gaze: A binary variable derived from eye offset, where gaze was coded as present (1) when the absolute offset angle was <25°, and absent (0) otherwise.; Facial expressions: Frame-level intensities (0–100) were computed for the following emotional categories: anger, disgust, fear, happiness, sadness, surprise, neutral, and positive (i.e. happiness) / negative affect (i.e. anger, disgust, fear, sadness) (3, 4).; Voice features: Speech segments were isolated from the videos, and a range of acoustic features were extracted using the *librosa* and *parselmouth* Python libraries (5, 6).

**Sample size calculation**

The target sample size was estimated using power analysis. Assuming that multiple regression would be used to predict manic or depressive symptoms from a combination of quantitative behavioral and clinical variables, with α = 0.05, power = 0.80, and an effect size of *f²* = 0.15 (medium), the required number of participants was estimated based on 3–7 predictors.

Approximately 80–100 cases were required for training data, and considering a 10% rate of missing data, the target sample size was set to 110. An additional 110 participants were allocated as test data, resulting in a minimum total sample size of 220.

However, since the analyses in this study were exploratory, and given that the number of predictors and analytical design could change, data collection continued beyond this minimum target to obtain as many cases as possible within the data collection period (until March 2024).

**Data Preprocessing**

All recorded data were pre-processed prior to analysis. Participants were divided according to their current or most recent depressive episode, and diagnostic classification (bipolar vs. unipolar depression) was determined based on the lifetime diagnosis obtained from SCID-5. Only participants with complete video and audio recordings were included in the analysis.

The dataset was randomly split into training, validation, and test subsets using ratios of 70%–15%–15% (or 75%–25% for smaller samples), while maintaining approximately equal distributions of target variables across splits. For the analysis of manic symptoms, excessive zero and low-end values in the YMRS distribution were handled through random subsampling: 60% of zero values and 20% of scores within the 1–6 range were randomly removed from the training set and evaluated separately. Potential outliers were also excluded to stabilize the model training.

**Feature Preparation**

A total of 42 behavioral and clinical features were used, including facial, vocal, and gaze-related indices, as well as sleep-related and clinical questionnaire measures. Multicollinearity among features was examined, and redundant variables were removed (|r| > 0.8).

Features with minimal variability (variance < 0.01) or very low correlation with target variables (r < 0.01) were excluded. The distributions and relationships between individual features and outcome variables were visually inspected using scatter and violin plots to guide feature retention decisions.

Feature selection was conducted via Recursive Feature Elimination with Cross-Validation (RFECV) using the Extreme Gradient Boosting (XGBoost) classifier, optimized for the mean F1 score across folds. Additional feature selection occurred during model training as part of the repeated cross-validation procedure. Multiple trials were run for each fold in the repeated cross-validation to optimize the objective function. Each trial represented a bagging classifier composed of various hyperparameter combinations and feature subsets. The model achieving the highest objective score in each fold was considered optimal.

**Model Training and Validation**

For the classification task differentiating unipolar and bipolar depression, bagging classifiers wrapped in a calibrated classifier (isotonic calibration) were trained using four-fold, three-times repeated cross-validation. The final prediction was made using a weighted ensemble model combining 12 bagging classifiers derived from cross-validation folds.

For the regression tasks predicting depressive or manic symptom severity, several machine learning models were trained, including CatBoost, ExtraTrees, RandomForest, and Ridge Regression. Hyperparameter tuning was performed using a composite metric defined as *RMSE / [1 + R²]* to balance bias and variance. A VotingRegressor ensemble was constructed by integrating the three best-performing models (and, in some cases, an additional Ridge model as a linear component).

Model performance was evaluated on training, validation, and unseen test datasets. Subgroup analyses were conducted stratified by sex and, for bipolar participants, by subtype (bipolar I vs. bipolar II).

**Model Evaluation and Interpretability**

Model performance metrics included accuracy, ROC-AUC, F1 score, sensitivity, specificity, positive predictive value (PPV), negative predictive value (NPV), R², and RMSE, depending on the analytic task.

Calibration of probabilistic outputs was evaluated using calibration plots, with model reliability assessed across probability bins.

Model interpretability was assessed using SHapley Additive exPlanations (SHAP) analysis. SHAP violin plots were used to visualize the relative contributions of individual features to model predictions. The mean absolute SHAP value (|SHAP|) was computed for each feature to quantify its global importance in the model. Permutation feature importance and SHAP analyses using the CatBoost model consistently identified the most influential predictors across tasks.

**Ground truth labels**

The ground truth labels correspond to the clinician-assigned scores recorded during the Zoom-based assessment sessions, specifically: the HAMD-17 (17-item Hamilton Depression Rating Scale) for depressive symptom severity, the YMRS (Young Mania Rating Scale) for manic symptom severity, and the SCID-5-RV diagnostic category for the bipolar versus unipolar classification task.

**Outlier exclusion**

As part of preprocessing, samples whose clinical profile presented as an observed abnormality relative to the rest of the dataset were flagged and excluded, since retaining such atypical cases risked introducing noise and disrupting the patterns the model needed to learn from the more representative cases. As an example of the type of case that fell under this criterion, one participant simultaneously showed a severe mania score (YMRS above 20) and a severe depression score, a combination that is typically more characteristic of bipolar disorder, while the clinical diagnosis recorded for this participant was unipolar depression. Therefore, we excluded cases of this kind as outliers. Separately, participants with low YMRS scores were strongly overrepresented in the dataset, which posed a risk of biasing the model toward low-score predictions. To address this, we performed a subsampling of the low-range YMRS values, as described below.

**YMRS subsampling**

The YMRS distribution in the dataset was strongly skewed toward low scores, because the majority of participants did not present with significant manic symptomatology. If the model is trained on this raw distribution, the simplest way for it to minimize training error is to predict values close to the low end for almost every input, since that strategy is correct for the majority of samples. The model therefore appears to learn well on the training data, but in reality it is learning the distribution rather than the relationship between behavioral features and symptom severity. The mid-to-high range, which is clinically the most important to identify, is underrepresented and the model receives too few examples there to learn the corresponding patterns. To rebalance the training distribution, based on previous literature (7, 8), we randomly removed 60% of the samples with a YMRS score of 0 and 20% of the samples with YMRS scores in the 1–6 range, which gives the model a meaningful signal across the full severity range. The samples removed from training were not discarded; they were retained as a separate held-out set so the model could still be evaluated on low-range YMRS values.

**References**

1.	Deng J, Guo J, Zhou Y, Yu J, Kotsia I, Zafeiriou S. (2019): RetinaFace: Single-stage Dense Face Localisation in the Wild. Arxiv.

2.	Wang Z, Chai J, Xia S. (2018): Realtime and Accurate 3D Eye Gaze Capture with DCNN-Based Iris and Pupil Segmentation. *IEEE Trans Vis Comput Graph* 27: 190-203.

3.	Zhang K, Zhang Z, Li Z, Qiao Y. (2016): Joint Face Detection and Alignment Using Multitask Cascaded Convolutional Networks. *IEEE Signal Process Lett* 23: 1499-1503.

4.	Serengil SI, Ozpinar A. (2021): HyperExtended LightFace: A Facial Attribute Analysis Framework. *2021 Int Conf Eng Emerg Technol (ICEET)* 00: 1-4.

5.	McFee B, Raffel C, Liang D, Ellis D, McVicar M, Battenberg E, *et al.* (2015): librosa: Audio and Music Signal Analysis in Python. *Proc 14th Python Sci Conf*: 18-24.

6.	Jadoul Y, Thompson B, Boer Bd. (2018): Introducing Parselmouth: A Python interface to Praat. *J Phonetics* 71: 1-15.

7.	Branco P, Torgo L, Ribeiro RP. (2019): Pre-processing approaches for imbalanced distributions in regression. *Neurocomputing* 343: 76–99.

8.	Liu XY, Wu J, Zhou ZH. (2009): Exploratory Undersampling for Class-Imbalance Learning. *IEEE Trans. Syst., Man, Cybern.Part B: Cybern.* 39: 539–550.

**Supplemental Figures**

**Supplemental Figure 1.** **Participants flow**

**Supplemental Figure 2.** **Trial scores for selected features.**

F1 scores of feature selection were presented for each trial. Multiple trials were run for each fold in the repeated cross-validation to optimize the objective function. Feature selection was conducted via Recursive Feature Elimination with Cross-Validation using the Extreme Gradient Boosting classifier, optimized for the mean F1 score across folds. Each trial represented a bagging classifier composed of various hyperparameter combinations and feature subsets. The model achieving the highest objective score in each fold was considered optimal.

**Supplemental Figure 3. Calibration for differentiation of bipolar from unipolar depression**

Calibration plots of weighted ensemble across probability bins were presented with perfect calibration line to assess model reliability. The curve generally aligns with the perfect calibration line, particularly in the mid- to high-probability ranges. However, some deviation is observed in the fifth bin (0.80–1.00). This bin contains only two data points, making it less reliable for evaluating perfect calibration. Despite the small sample size, the fraction of positives is slightly underpredicted. Overall, the model demonstrates improved calibration as the predicted probabilities increase, particularly from the mid- to high-confidence bins.

| **Supplemental Table 1. Future list and their explanations** | |
| --- | --- |
| **Feature Name** | **Explanation** |
| AQ | Autism quotient score |
| RMS | Root mean square: Reflects the loudness or energy of the voice (dbs) |
| Pitch_m | Mean pitch of speech |
| Pitch_s | Standard deviation of pitch; Captures pitch variability. |
| Zero_crossing | Zero crossing measures how often a person’s voice signal changes direction. Higher values can make the voice sound more tense or agitated, while lower values suggest a calmer, smoother tone. |
| Tempo | Speed or rhythm of speech delivery |
| HNR | Harmonic to Noise ratio; Measure of how clear the voice is as compared to the unwanted noise |
| Local_Jitter | Local jitter measures how much the pitch of someone’s voice slightly wobbles from one moment to the next. It shows how steady or shaky the voice is. A higher value means the voice may sound rough, hoarse, or unsteady. |
| Local_Shimmer | Local shimmer measures tiny changes in the loudness of someone’s voice as they speak. It reflects how stable the voice’s volume is. A higher value means the voice may sound breathy, weak, or uneven. |
| Blink_m | Mean eye blink rate. |
| Angry_s | Standard Deviation of the angry facial expression |
| Fear_s | Standard Deviation of the fearful facial expression |
| Happy_s | Standard Deviation of the happy facial expression |
| Sad_s | Standard Deviation of the sad facial expression |
| Neutral_s | Standard Deviation of the neutral facial expression |
| Negative_s | Standard Deviation of the negative (Angry + Disgust + Fear + Sad) facial expression |
| Blink_s | Standard Deviation of the eye blink |
| Gaze_s | Standard Deviation of the eye gaze (1 if absolute of eye_offset is less than 25 degrees) |
| Eye_offset_s | Standard Deviation of the angle from the camera |
| Angry_ss | Standard Deviation of Standard Deviation (Standard deviation for every 30 seconds is calculated and then the standard deviation of this calculated standard deviation is evaluated.) of the Angry facial expression |
| Fear_ss | Standard Deviation of Standard Deviation of the fearful facial expression |
| Happy_ss | Standard Deviation of Standard Deviation of the happy facial expression |
| Sad_ss | Standard Deviation of Standard Deviation of the sad facial expression |
| Neutral_ss | Standard Deviation of Standard Deviation of the neutral facial expression |
| Negative_ss | Standard Deviation of Standard Deviation of the negative facial expression |
| Blink_ss | Standard Deviation of Standard Deviation of the eye blink |
| Gaze_ss | Standard Deviation of Standard Deviation of the eye gaze |
| Eye_offset_ss | Standard Deviation of Standard Deviation of the eye offset |
| Sleep_efficiency_recent | Calculated as the average sleep time over the past week divided by the total time spent in bed during the same period. This metric represents how efficiently the person slept over the last week. |
| Sleep_efficiency_usual | Calculated as the usual average sleep time divided by the usual total time spent in bed. This metric represents how efficiently the person typically sleeps. |
| Change_in_sleep_efficiency | Calculated as sleep_efficiency_recent minus sleep_efficiency_usual. This indicates how recent sleep efficiency differs from the individual’s usual pattern. |
| Change_in_sleep-onset_latency | Calculated as the time taken to fall asleep over the past week minus the usual time to fall asleep. It measures the change in sleep-onset latency compared with the usual pattern. |
| Change_in_bed_time | Calculated as the recent total time spent in bed minus the usual total time spent in bed. This reflects how total time spent in bed has changed over the past week. |
| Change_in_sleep_time | Calculated as the average sleep time over the past week minus the usual average sleep time. It reflects the change in actual sleep duration compared with the typical sleep pattern. |
| Psychosis_Diagnosis | A combined score based on the current diagnosis of schizophrenia spectrum and psychotic disorders. |
| Mood_Diagnosis | A total score based on the current diagnosis of mood disorders. |
| Substance_Diagnosis | An aggregated score based on the current diagnosis of substance-related and addictive disorders. |
| Anxiety_Diagnosis | A combined score derived from the current diagnosis of anxiety disorders. |
| OCD_Diagnosis | An aggregated score based on the current diagnosis of obsessive compulsive and related disorders. |
| Eating_Diagnosis | A combined score based on the current diagnosis of feeding and eating disorders. |
| Trauma_Diagnosis | An aggregated score based on the current diagnosis of trauma and stressor-related disorders. |
| Antisocial_Diagnosis | A score based on the current diagnosis of Anti-social personality disorder. |
